# Effectiveness of Fluoride Varnish Application as Cariostatic and Desensitizing Agent in Irradiated Head and Neck Cancer Patients

**DOI:** 10.1155/2013/824982

**Published:** 2013-06-13

**Authors:** Kanchan P. Dholam, Priyanka Piyush Somani, Seema D. Prabhu, Shubhangi R. Ambre

**Affiliations:** Dental and Prosthetic Surgery, Tata Memorial Hospital, Dr. E Borges Road, Parel, Mumbai 400 012, India

## Abstract

*Objective*. To evaluate the effectiveness of three-month fluoride varnish application on radiation caries and dental sensitivity and to assess compliance to three-month fluoride varnish application. *Materials and Methods*. 190 irradiated head and neck cancer patients were randomly selected and reviewed retrospectively. Oral prophylaxis, fluoride varnish application, and treatment of dental caries were done prior to radiation therapy. Patients were followed up at every three months for dental evaluation and fluoride varnish application. Decayed-missing-filling-teeth indices, dental sensitivity, and compliance to fluoride varnish application were noted for fifteen months and analyzed statistically. *Results*. Significant increase in decayed-missing-filling-teeth index was seen at nine (*P* = 0.028), twelve (*P* = 0.003) and fifteen (*P* = 0.002) months follow-up. However, the rate of increase in decayed-missing-filling-teeth indices was 1.64/month which is less than the rate mentioned in the literature (2.5/month). There was no significant effect of sex (*P* = 0.952) and surgery (*P* = 0.672) on radiation caries, but site of disease (*P* = 0.038) and radiation dose (*P* = 0.015) were found to have statistically significant effect. Dental sensitivity decreased from 39% at 3 months to 25% at 15 months followup. 99% compliance to fluoride varnish application was seen till six months followup which decreased to 46% at fifteen months. *Conclusion*. Three-month fluoride varnish application is effective in decreasing radiation caries and sensitivity and has good compliance.

## 1. Introduction

Radiation therapy plays an important role in the management of patients with head and neck cancer. It is also associated with several undesired reactions. In the long term, the irradiated patients are susceptible to atrophy and fibrosis of the muscles of mastication that can lead to trismus and xerostomia leading to extensive dental caries and osteoradionecrosis [1].

Radiation caries is a specific form of dental caries with multifactorial etiology. It is highly destructive with a rapid onset, progression, and nonspecific localization. Xerostomia is the main risk factor [2]. An ideal approach to prevent radiation caries is quantitative and qualitative modification of saliva [1]. Radiation-induced hyposalivation can be avoided by excluding major and minor salivary glands from irradiation field [3]. 

Topically applied fluorides buffers pH of saliva reduces oral cariogenic flora and remineralises tooth structure there by qualitatively altering the saliva [4]. As desensitizing agents, fluorides work by blocking the dentinal tubules and prevent the movement of fluid backward and forward within the dentinal tubules in response to stimuli of pain [5].

Radiated head and neck cancer patients are high risk patients for dental caries and dental sensitivity. As per American Dental Association (ADA) recommendations for high risk patients, the fluoride varnish should be applied 2–4 times per year. The fluoride varnish has been found to be effective in preventing caries in high risk patients [6].

The successful use of topically applied fluorides to prevent radiation caries has been described by several authors. Daly et al. [7] and Dreizen et al. [8] reported the use of 1.0% neutral NaF, or NaF_2_ gel (4,500 parts per million fluoride ion), applied daily in custom tray. However, the compliance with fluoride application in carriers by the population of patients with head and neck cancer is generally thought to be poor [9, 10].

 The aim of this research was to determine the effect of fluoride varnish application on radiation caries and dental sensitivity in head and neck cancer patients. The intention was also to assess the compliance of patients to three monthly fluoride varnish application (FVA).

## 2. Materials and Methods

Head and neck cancers are one of the most prevalent cancers in India in view of the social habits. Almost all of these patients receive definitive or adjuvant radiation therapy as part of the treatment. 

 A complete clinical and radiological oral examination is considered as an integral component of overall medical care at our institute before initiation of radiation therapy. Existing decayed teeth are salvaged, if possible, or extracted. Thorough oral prophylaxis, followed by application of slow release aqueous-based topical 5% NaF varnish (Fluoritop-SR, ICPA Health Products Ltd. Mumbai, India), is done. Detailed oral hygiene instructions are given to the patients. These patients are followed up every three months for dental evaluation and FVA by single clinician. 

190 head and neck cancer patients, who received radiation therapy, were randomly selected and reviewed. These patients had completed clinical and radiological oral examinations before initiation of radiation therapy. All these patients had undergone thorough oral prophylaxis, and FVA was done prior to radiation therapy and at every three months follow-up. Preradiotherapy, followup decayed-missing-filled-teeth Index (DMFT), dental sensitivity, and compliance to three-month FVA were recorded till fifteen months followup.

Patients' demographics, tumor location, staging, histopathology, radiation dosage, and surgery were recorded. All these patients were treated with conventional 2D radiation therapy technique. The patients were divided into three groups depending on their radiation dose, namely, Group 1: <50 Gy, Group 2: 50–60 Gy, and Group 3: >60 Gy. Statistical calculations were performed by using Mann-Whitney *U* test or Kruskal-Wallis test (as appropriate) for continuous variables and chi-square test or Fisher's exact test for categorical variables. All of the tests were conducted at 5% level of significance (2-sided). The data was entered in SPSS v18 software and was analyzed statistically using repeated measures ANOVA (with Bonferroni Correction). Numeric data is represented as mean ± standard deviation or frequency. 

## 3. Results

A total of 190 patients were included in the study with 138 males and 52 females. The mean age of patients was 46.5 years (SD = 13.5, range from 16 to 77 years). The diagnosis (histopathology and site) is shown in Tables 1 and 2. The patients had been treated with 2D external beam radiation therapy to the mean dose of 58.4 Gy (SD = 8.3, range = 20–75 Gy). The majority of the patients, 71.2%, had received combination therapy of surgery and radiation therapy with or without chemotherapy. 28.2% of patients were treated with radiation therapy alone. 10 patients were treated with total dose less than 50 Gy, 95 patients received radiation dose between 50 and 60 Gy, and 36 were delivered a dose higher than 60 Gy. Radiation dose for 49 patients could not be retrieved from the records.

Preradiotherapy mean of initial DMFT for patients was 4.1 ± 4.3 (Table 3). With the three-month FVA, the mean DMFT increased to 5.1 ± 5.4 at fifteen months follow-up visit. The caries incremental rate was (a) preradiotherapy to six months—1.3/month (8.02%), (b) six months to fifteen months—1.7/month (15.35%), and (c) preradiotherapy to fifteen months—1.6/month (24.6%). 

Statistically significant increase in DMFT index was seen at nine (*P* = 0.03), twelve (*P* = 0.003), and fifteen (*P* = 0.002) months indicating that the progression of radiation caries is a late effect of radiation therapy (Table 3). When the data was compared for DMFT across the study period with respect to sex of the patient or surgery as an additional treatment modality, it was seen that sex (*P* = 0.952) and surgery (*P* = 0.107) had no significant effect on radiation caries, but the site of the disease (*P* = 0.038), and radiation dose (*P* = 0.015) were found to have statistically significant effect. Due to wide variation in the number of patients for different sites and different radiation dose groups, the significance for DMFT index with the study period was not done.

**Figure 1:**
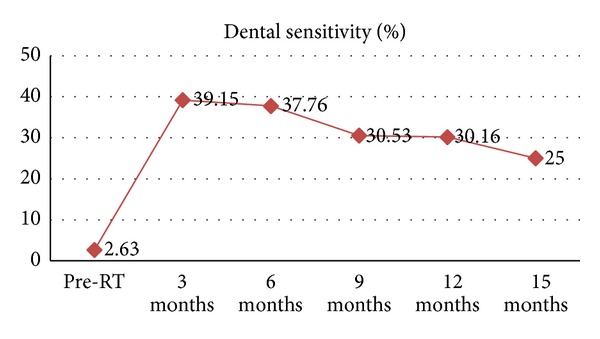
Graph showing dental sensitivity at each followdup visit after FVA.

Only 2.6% of patients had preradiotherapy dental sensitivity. This increased to 39% at three-month followup. Though the increase in sensitivity was highly significant (*P* < 0.001) at each follow-up visit when compared to base line, it was not significant when compared between consecutive follow-up visits. Significant decrease in sensitivity was seen between twelve and fifteen months follow-up visits (*P* = 0.003). With the regular FVA, sensitivity decreased to 25% at fifteen-month followup (Figure 1).

**Figure 2:**
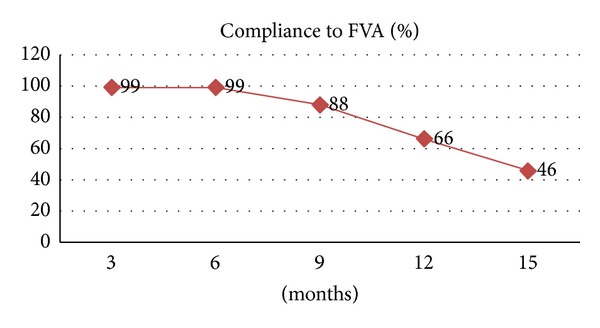
Graph showing compliance of the patient to FVA.

Compliance to FVA was calculated with patient's follow-up visit. It was observed that the compliance was good till nine-month followup and gradually decreased thereafter till fifteen months (Figure 2).

## 4. Discussion

Preventive measures for radiation caries before, during, and after radiotherapy are necessary and should include instructions regarding a noncariogenic diet, thorough regular oral hygiene, and application of fluoride. Though consensus exists regarding fluoride application in these patients, controversy persists about type of fluoride, frequency, concentration, and method of fluoride application due to a lack of fundamental research in this field [11]. 

As per the author's knowledge, there are no studies in the literature where the dental caries index and effectiveness or compliance of professionally applied fluoride varnish at three-month interval are studied in Indian head and neck cancer patients. 

Dreizan et al. [8] studied the incidence of radiation caries in patients with three different caries protective regimens, that is, plaque-disclosing dye: non fluoride gel, unrestricted diet, plaque-disclosing dye: fluoride gel, unrestricted diet and plaque-disclosing dye: fluoride gel, sucrose-restricted diet. They found that the mean caries incremental rate was 1.3/month in Group I. In Group II, patients who were on 1% sodium fluoride regimen with plastic gel carriers at three-month interval for first postradiation year and at six-month interval thereafter, the mean caries increment rate was 0.07/month. 0.03 mean caries incremental rate per month was seen in Group III.

In Bosnian population, Konjhodzic-Prcic et al. [2] reported caries incremental rate of 3.5/month for irradiated head and neck cancer patients. However, there was no caries protective regimen mentioned.

In our study with the three-month FVA, the caries incremental rate was found to be 1.6/month for fifteen months. However, it was seen that the rate of caries progression was slower initially, till first six months, that is, 1.3/month. The caries incremental rate increased after six months to 1.7/month. The rate of increase in radiation caries was less than that mentioned in the literature, 2.5/month [7, 12].

 Due to unequal distribution of patients in different sites (Table 1) and different radiation dose group, though the site (*P* = 0.038) and radiation dose (*P* = 0.015) showed statistically significant effect on DMFT (*P* = 0.038), no inference could be obtained for site specificity, radiation dose and DMFT. 

It has also been stated that during and following radiotherapy, the teeth may become hypersensitive, which could be related to the decreased secretion of saliva and the lowered pH of secreted saliva. The topical application of a fluoride relieves these symptoms [13]. Studies have found that topical fluoride application forms fairly insoluble globules like calcium fluoride-like material on the tooth surface. These globules act as a reservoir of fluoride and block dentinal tubules thereby reducing dental sensitivity [14]. 

The retrospective analysis of the data in this study supports the documentation made in the literature regarding radiation induced dental sensitivity and fluoride treatment for the same. It was observed that the radiation sensitivity increased significantly by 36.5% at three-month followup after radiation therapy. With the regular FVA, it was seen that radiation sensitivity decreased to 25% at fifteen-month followup.

In a systemic review by Marinho [15], the assessment of effectiveness of fluoride toothpastes, gels, varnishes, and mouth rinses through comparisons against nonfluoride controls, against each other, and against different combinations is done. The average decayed-missing-filled-surface indices prevented fraction that is, percentage caries reduction ranged from 24% for fluoride toothpaste to 26% for mouth rinses and 28% for gels to 46% for fluoride varnishes.

The long-term compliance with the use of fluoride gel in custom tray has been reported as very poor by Carl [9], Boyett [16] (57% noncompliant), and Daly et al. [7] (85% fair to poor compliant). The explanation given by Jansma et al. [17] was that the use of tray was inconvenient and time consuming for patients. Shannon and Edmonds [18] considered painful mucositis following radiotherapy very discouraging to the performance of oral hygiene procedures. Joyston-Bechal et al. [19] supported the fact that the cancer patients are often depressed to concentrate on prophylactic dental procedures. Lockhart and Clark [20] reported the gagging with trays and sociological factors leading to lack of concern for general health and hence additional cause for poor compliance.

It has been reported that the fluoride varnish is easy to apply, creates less patient discomfort, achieves greater patient acceptability, and has lesser toxicity than fluoride gel. Quantity of fluoride in varnish is less than the gel thus reduces the risk of inadvertent ingestion. However, to our knowledge, there are no articles in the literature with the assessment of compliance to three-month FVA in radiated head and neck cancer patients.

Due to lack of data in the literature and in the view of the advantages of fluoride varnish over gels, compliance to the three-month FVA protocol is assessed. It is observed that the compliance was almost 100% till six months. Poor long-term compliance of these patients can be attributed to long distance travelling, financial constraints, progression of disease, death due to disease, or other reasons. Though there was a definite decrease in compliance, it was still better than the compliance stated in the literature for fluoride gel application.

## 5. Limitations

This is a retrospective study, thus, there is a lack of local historical controls or concurrent controls to allow any conclusion on the impact of flouride varnish on caries in HNC patients. Xerostomia, diet frequency, sucrose intake, and so forth, which are recognized as risk factors in these patients, are not documented. Prospective studies can be conducted to assess the correlation of xerostomia, fluoride varnish application, diet, and dental caries in head and neck cancer patients.

Also, the data of noncompliant patient for DMFT index and dental sensitivity was not recorded. Thus, the potential effect of fluoride in the compliant versus noncompliant population could not be assessed, though there is a dichotomy in compliance with varnish application.

## 6. Conclusion

Radiation caries is the late effect of radiotherapy. Three-month FVA helps in decreasing the incidence of radiation caries. It also helps in decreasing the radiation induced dental sensitivity. Though the compliance with three-month FVA is better than fluoride gel in custom carrier, there is still a need to educate these patients about the need of FVA. Future studies with larger sample and near equal distribution of patients for specific sites and different radiation dose are required to know the effect of site specificity and radiation dose on radiation caries. Continuing studies comparing different methods of topical fluoride application in head and neck cancer patients can guide the clinician for selection of better topical fluoride.

## Figures and Tables

**Table 1:** Site of primary tumor (*n* = 190 patients).

Site of primary tumor	No.
Oral cavity	108
Oropharynx	35
Salivary glands	12
Para nasal sinus	20
Others (larynx, nasopharynx, and unknown primary)	33

**Table 2:** Type of primary tumor (*n* = 190 patients).

Type of primary tumor	No.
Squamous cell carcinoma	156
Salivary gland tumor	15
Undifferentiated carcinoma	04
Others	15

**Table 3:** Statistical evaluation of DMFT.

	Before RT	3 months	6 months	9 months	12 months	15 months
Mean	4.12	4.28	4.46	4.83	5.04	5.14
SD	4.35	4.42	4.85	5.15	5.31	5.36
Max.	32	32	32	32	32	32
Min.	0	0	0	0	0	0
*P* value		0.188	0.725	0.028	0.003	0.002
